# Increased risk of hepatitis B virus infection amongst individuals with diabetes mellitus

**DOI:** 10.1042/BSR20181715

**Published:** 2019-03-29

**Authors:** Xuan Zhang, Xia Zhu, Yulin Ji, Hong Li, Fengsu Hou, Chuan Xiao, Ping Yuan

**Affiliations:** 1 Department of Epidemiology and Statistics, West China School of Public Health, Sichuan University, Chengdu 610041, Sichuan Province, China; 2 Center of Infectious Disease, West China Hospital, Sichuan University, Chengdu 610041, Sichuan Province, China; 3 Department of Respiratory and Critical Care Medicine, West China Hospital, Sichuan University, Chengdu 610041, Sichuan Province, China; 4 Department of Urology, West China Hospital, Sichuan University, Chengdu 610041, Sichuan Province, China

**Keywords:** Blood glucose monitoring, Diabetes mellitus, Hepatitis B virus, Prevention, Risk of infection

## Abstract

There have been reports of hepatitis B outbreaks amongst diabetics in long-term care facilities, suggesting that risk of hepatitis B virus (HBV) infection is higher in this population. However, the magnitude of the risk and the incidence of HBV infection amongst the general diabetic population in China remains unknown. Data from a cohort study conducted in Mianyang City, Sichuan Province, China, were retrospectively analyzed in order to address this question. Demographic information was collected using a custom-designed questionnaire, and blood samples were tested for HBV using ELISA. We used multivariate logistic regression to explore the relationship between HBV infection and diabetes, while adjusting for age, sex, region, medical insurance, exposure history, and HBV vaccination. During 2013–2014, a total of 189766 adults were surveyed, of which 7382 were newly infected with HBV, corresponding to an incidence of 3.89%. In this study population, there were 4982 diabetic patients and 182710 non-diabetic individuals. Amongst those with diabetes, 265 (5.32%) were newly infected with HBV. In contrast, 7038 (3.85%) in the non-diabetic population were newly infected with HBV. The relative risk (RR) of HBV infection was 43% higher amongst those diagnosed with diabetes than amongst those not diagnosed (RR 1.43, 95% confidence interval (CI) 1.26–1.63). These results suggest that the risk of HBV infection is higher amongst individuals diagnosed with diabetes mellitus in Mianyang City, Sichuan Province, China. Hepatitis B vaccination and continuous infection control practices may help to reduce HBV infection in diabetic patients, and should be considered for diabetes management.

## Introduction

Hepatitis B virus (HBV) is a serious and prevalent health problem: an estimated 257 million people live with HBV infection worldwide [1]. HBV is endemic in China, where there are 93 million hepatitis B surface antigen (HBsAg) carriers and 25 million chronically infected patients, according to the most recent national HBV seroprevalence data from 2006 [2,3]. HBV infection is associated with substantial health risks, since approximately 5% of adults with acute or asymptomatic HBV infection become chronically infected, and individuals infected with this virus are at increased risk of developing hepatic decompensation, cirrhosis, and hepatocellular carcinoma [4,5]. Routes of HBV transmission include mother-to-child, blood-borne, and sexual transmission [1,6]. HBV can spread when medical instruments and syringes are not properly disinfected, during invasive treatments and surgical operations, and as a result of intravenous drug abuse [5]. The most likely route of spread is percutaneous or mucosal exposure to blood or body fluids from infected patients and carriers [4]. In addition, HBV can remain stable on environmental surfaces for more than 7 days [7,8].

China has the largest number of adult diabetic patients worldwide, who require comprehensive medical care to manage blood glucose and to prevent diabetes complications such as cardiovascular disease, kidney disease, retinopathy, and neuropathy [9,10]. Regardless of whether patients with diabetes are receiving insulin, oral agents, or dietary therapy, 88% must monitor their blood glucose levels at least once monthly [11]. Assisted monitoring takes place in various venues, including physician offices, hospitals, health fairs, schools, and assisted-living facilities [12]. HBV transmission can occur when patients are exposed to blood or body fluids from infected people through contaminated equipment or surfaces (e.g., through blood glucose monitoring equipment, when insulin pens are used for more than one individual, or during certain procedures) [12–16]. In addition, patients with diabetes mellitus have altered levels of T-lymphocyte subsets that suggest defective cellular immune function, and therefore might be at higher risk of infection than adults with normal immune function [17–19].

Several studies have described hepatitis B outbreaks amongst people with diabetes attending long-term care facilities, such as nursing homes and assisted-living facilities, suggesting an increased risk of HBV infection in these environments [14,20–25]. However, the risk and incidence of HBV infection amongst diabetic patients in China remains unknown. Therefore, we aimed to evaluate the risk of HBV infection amongst individuals diagnosed with diabetes mellitus in Mianyang City, Sichuan Province, China.

## Materials and methods

### Ethical approval

The present study was approved by the Ethics Committee of West China Hospital, Sichuan University, and was performed in agreement with the Declaration of Helsinki. Each participant signed an informed consent form before enrollment.

### Study population

As part of ‘The National Science & Technology Pillar Program during the 12th Five-year Plan Period’, implemented from June 2013 to March 2014, data were collected on HBV infection in the general population of Mianyang, the second largest city of Sichuan Province with a population of 48109000. We applied stratified random cluster sampling to recruit participants. First, we stratified the area into two levels based on gross domestic product (GDP) data from the Mianyang Bureau of Statistics. In each of the two levels, we randomly selected one administrative division (Fucheng District and Jiangyou City). Second, we stratified towns/subdistricts in these two divisions into three levels based on GDP data. In each of the three levels, we randomly selected 7 towns/subdistricts, i.e. 10 towns/subdistricts from Fucheng District and 11 from Jiangyou City. Third, within each town/subdistrict, we selected all villages or residential communities. Finally, we used proportional random sampling to select 60% of local residents in each village or residential community as the study population.

Individuals identified in this way were included in the study if they satisfied the following criteria: (i) they had lived in the sampled community for more than 6 months at the time of study enrollment; (ii) they were negative for HBsAg based on a serologic test conducted in 2009 as part of physical examinations performed under the 11th Five-year Plan; (iii) they were at least 15 years old, since younger individuals were most likely to have been vaccinated [5]; and (iv) they participated in the physical examination of the 12th Five-year Plan.

### Data collection

Research staff received appropriate training and then collected data at examination centers in local health facilities and community clinics located in the towns/subdistricts selected for inclusion in the study. The staff conducted standardized face-to-face interviews via questionnaires after obtaining written consent from participants. The questionnaire collected demographic information (including sex, age, and area of residence), information from medical history (including diabetes, hypertension, family history of hepatitis B, blood transfusion, and surgical interventions), and payment method (including medical insurance payment and self payment). Individuals with medical insurance may visit medical services more often, and therefore it is plausible that they are at higher risk of opportunistic infection [26]. Each questionnaire was assigned a unique identification number.

After the interview, 5 ml fasting blood sample was collected from each participant, and labeled with the same identification number as the questionnaire. Blood samples were stored at 4–8°C and transported within 1 day to clinical laboratories for processing and serological testing. HBsAg was assayed using a commercial enzyme-linked immunosorbent assay (ELISA) kit (480T, Xinhaiwan Company, Chengdu, China). Fasting blood glucose was measured using the glucose oxidase/peroxidase assay kit (200 ml, Yuanhehuasheng Company, Chengdu, China).

Completed questionnaires were scanned as images, and then optical character recognition technology was used to convert images into digital data, which were stored using a special software developed by technicians from the College of Computer Science from Sichuan University. Biochemical data were directly exported and stored using the software. Finally, all these data were exported directly into a Microsoft Excel database.

### Definitions

Diabetic individuals were defined as those who responded ‘yes’ to the following question: ‘Other than during pregnancy, have you ever been told by a doctor or health professional that you have diabetes or diabetes mellitus, and are you currently being managed for chronic disease?’. All individuals with diabetes met World Health Organization diagnostic criteria [27]. People who responded that they were borderline diabetic were considered non-diabetic. If the patient could not fill out the questionnaire, information was obtained from physician or medical records. If none of these sources was available, diabetes status was classified as unknown.

Identification and recordkeeping on diabetic individuals was expected to be fairly reliable, since as part of the ‘National Basic Public Health Services’ concept implemented in China since 2009, each township hospital maintains and annually updates records on all individuals with chronic disease, such as hypertension and diabetes. Hospitals provide chronic disease management to such individuals, including blood glucose monitoring at least once a month and follow-up services. These individual records were accessible through a centralized database.

Newly HBV-infected patients were defined as those who were negative for HBsAg in 2009, when they participated in the physical examination of the 11th Five-year Plan, but who were positive for HBsAg during the examination of the 12th Five-year Plan and who did not report history of HBV. We assumed that no subjects were infected with HBV at the start of the study.

### Quality control

The Center for Disease Control and Prevention of Mianyang City distributed questionnaires as well as equipment and supplies for blood collection, serum separation, and serum preservation to community health service centers and township hospitals in the study. All serum samples testing positive for HBsAg were retested at the Center for Disease Control and Prevention of Mianyang City.

### Statistical analysis

Demographic information included age, sex, and region. We divided age into two groups, 15–59 and ≥60 years old, since older adults are more likely to receive medical service and show decreased responsiveness to HBV [28]. History of exposure to HBV infection was a composite variable determined by history of surgery, trauma, and blood transfusion, as well as family history of HBV infection. Diabetic and non-diabetic patients were compared in terms of age, sex, region, exposure history, HBV status, and history of HBV vaccination. Individuals were divided into three groups based on fasting blood glucose: <7.0, 7.0–8.0, and >8.0 mmol/l [29]. However, patients with fasting blood glucose between 7 and 8 mmol/l were relatively rare in this study. Newly infected individuals were stratified according to diabetes status, demographics, medical insurance, exposure history, and history of HBV vaccination, and the subgroups were compared using chi-squared tests.

We applied multivariate logistic regression to investigate the association between diabetes and HBV infection while adjusting for several confounding factors, including age, sex, region, exposure history, medical insurance, and history of HBV vaccination. Sensitivity analyses were performed by assuming that patients of unknown diabetes status did not have diabetes (Model 1) or did (Model 2).

Data were analyzed using SPSS 20.0 (IBM, Armonk, NY, U.S.A.) at the Department of Epidemiology and Statistics of West China School of Public Health at Sichuan University. The threshold of significance was defined as *P*<0.05.

## Results

### Characteristics of the study participants

In total, 189766 individuals (80115 males) with a mean age of 47.8 ± 17.7 years (range 15–97 years) were enrolled in the present study, of whom 7382 were newly infected with HBV during 2013–2014, corresponding to an incidence of 3.89%. Jiangyou City accounted for a higher proportion of subjects than Fucheng District (53.7 compared with 44.8%, respectively). Exposure history was reported by 21.2% of participants, and 19.1% had received at least one dose of the HBV vaccine.

There were 4982 diabetic patients in the study population. Amongst diabetic patients, 265 (5.32%) were newly infected with HBV. Amongst people diagnosed without diabetes, 7038 (3.85%) were newly infected (*P*<0.001, Table 1). The mean age of diabetic patients was 62.8 ± 9.8 years (range: 15–89 years), and the mean age of non-diabetic individuals was 47.3 ± 17.7 years (range: 15–97 years). Diabetes was more frequent amongst female than male subjects (61.0 compared with 39.0%) and more frequent in Jiangyou City than in Fucheng District (54.2 compared with 44.6%). History of exposure to HBV infection was higher in diabetic patients than in non-diabetic individuals (34.7 compared with 20.7%, *P*<0.001), although a greater proportion of non-diabetic individuals had received at least one dose of HBV vaccine (19.5 compared with 7.0%, *P*<0.001). Mean fasting blood glucose was 8.65 ± 2.01 mmol/l amongst diabetic individuals and 5.29 ± 2.32 mmol/l amongst non-diabetic individuals (Table 1).

**Table 1:** Characteristics of the study population

Characteristics		Diabetics, *n*=4982	Non-diabetics, *n*=182710	χ^2^	*P*
Age, years	15–59	1161 (23.3)	130883 (71.6)		
	≥60	3371 (67.7)	51827 (28.4)	3667.629	<0.001
	*Mean ± S.D.*	*62.8 ± 9.8*	*47.3 ± 17.7*		
Sex	Male	1944 (39.0)	77328 (42.3)	23.804	<0.001
	Female	3038 (61.0)	105382 (57.7)		
Region	Jiangyou	2702 (54.2)	97684 (53.5)	417.794	<0.001
	Fucheng	2222 (44.6)	82634 (45.2)		
History					
HBV exposure^1^	Yes	1729 (34.7)	37853 (20.7)	640.937	<0.001
	No	3223 (64.7)	143064 (78.3)		
HBV vaccination	Yes	349 (7.0)	35572 (19.5)	722.404	<0.001
	No	3392 (68.1)	123892 (67.8)		
HBV infection	Yes	265 (5.3)	7038 (3.8)	27.958	<0.001
	No	4717 (94.7)	175672 (96.2)		
Fasting blood-glucose, mmol/l	<7.0	1830 (36.7)	147898 (80.9)	434.101	<0.001
	7.0–8.0	672 (13.5)	3443 (1.9)		
	>8.0	2480 (49.8)	3332 (1.8)		
	*Mean ± S.D.*	*8.65 ± 2.01*	*5.29 ± 2.32*		

Values are n (%) or mean ± SD. Percentages may not add up to 100 due to missing data.

1 History of HBV exposure was a composite variable determined by history of surgery, trauma, and blood transfusion, as well as family history of HBV infection.

### Characteristics of newly HBV-infected patients stratified by diabetes status

Amongst newly infected patients, those with diabetes were older than those without diabetes (61.8 ± 9.4 compared with 49.4 ± 14.5 years, *P*<0.001; Table 2). The proportion of female subjects was higher amongst those with diabetes than amongst non-diabetics (60.4 compared with 50.0%, *P*<0.05). Fasting blood glucose was significantly higher amongst individuals with diabetes than amongst those without diabetes (8.37 ± 1.96 compared with 5.34 ± 1.97 mmol/l, *P*<0.001). Amongst newly infected patients, those with diabetes were less likely to have been vaccinated for HBV than those without diabetes (6.0 compared with 11.1%, *P*<0.05). Patients with diabetes were more likely to have a history of surgery than those without diabetes (34.0 compared with 18.1%, *P*<0.001), as well as a history of exposure to HBV infection (37.7 compared with 20.5%, *P*<0.001). The two subgroups were similar in history of trauma, family history of HBV infection, region, or payment method.

**Table 2:** Demographics and HBV exposure history for newly HBV-infected patients, stratified by diabetes status

Characteristics		Diagnosis of diabetes mellitus			χ^2^	*P*
		Yes (*n*=265)	No (*n*=7038)	Unknown (*n*=79)		
Age, years	15–59	99 (37.4)	5164 (73.4)	51 (64.6)	164.574	<0.001
	≥60	166 (62.3)	1874 (26.6)	28 (35.4)		
	*Mean ± S.D.*	*61.8 ± 9.4*	49.4 ± 14.5	*54.2 ± 9.8*	——	——
Sex	Male	105 (39.6)	3516 (50.0)	34 (43.0)	10.911	0.001
	Female	160 (60.4)	3522 (50.0)	45 (57.0)		
Fasting blood glucose, mmol/l	<7.0	101 (38.1)	5317 (75.5)	70 (88.6)	397.968	<0.001
	7.0–8.0	63 (23.8)	287 (4.1)	0 (0.0)		
	>8.0	101 (38.1)	454 (6.5)	3 (3.4)		
	*Mean ± S.D.*	*8.37 ± 2.01*	*5.34 ± 2.32*	*5.85±1.96*		
History						
HBV	Yes	100 (37.7)	1440 (20.5)	24 (30.4)	45.571	<0.001
exposure^1^	No	163 (61.5)	5529 (78.6)	54 (68.4)		
HBV	Yes	16 (6.0)	778 (11.1)	2 (2.5)		
vaccination	No	212 (80.0)	5252 (74.6)	58 (73.4)	6.867	0.009
Surgery	Yes	90 (34.0)	1272 (18.1)	17 (21.5)	42.496	<0.001
	No	175 (66.0)	5766 (81.9)	62 (78.5)		
Trauma	Yes	11 (4.2)	219 (3.1)	7 (8.9)	0.904	0.342
	No	254 (95.8)	6819 (96.9)	72 (91.1)		
Transfusion	Yes	7 (2.6)	101 (1.4)	3 (3.8)	2.551	0.110
	No	258 (97.4)	6937 (98.6)	76 (96.2)		
Hepatitis B in family	Yes	4 (1.5)	108 (1.5)	5 (6.3)	0.000	0.986
	No	256 (96.6)	6850 (97.3)	72 (91.1)		
Region	Jiangyou	137 (51.7)	3433 (48.8)	63 (79.8)	0.903	0.342
	Fucheng	124 (46.8)	3503 (49.8)	16 (20.2)		
Payment method	Medical insurance payment	262 (98.9)	6926 (98.4)	78 (98.7)	0.348	0.555
	Self payment	3 (1.1)	112 (1.6)	1 (1.3)		

Values are *n* (%) or mean ± S.D. Percentages may not add up to 100 due to missing data.

1 History of HBV exposure was a composite variable determined by history of surgery, trauma, and blood transfusion, as well as family history of HBV infection.

### Multivariate logistic regression

After adjusting for several potential confounding factors, including age, sex, region, medical insurance, history of exposure to HBV infection, and history of HBV vaccination, we identified having diabetes as positively associated with HBV infection (relative risk (RR) 1.43, 95% confidence interval (CI) 1.26–1.63; Table 3).

**Table 3:** Multivariate logistic regression to identify covariates associated with new HBV infection in the study population

Characteristics		RR	95% CI
Age, years	15–59	——	——
	≥60	0.80	0.76–0.84
Sex	Female	——	——
	Male	1.38	1.32–1.45
Region	Fucheng	——	——
	Jiangyou	0.81	0.78–0.85
Payment method	Self-payment	——	——
	Medical insurance payment	1.26	1.03–1.53
History of HBV vaccination	No	——	——
	Yes	0.47	0.43–0.51
Diabetes	No	1	——
	Yes	1.43	1.26–1.63
Fasting blood glucose, mmol/l	<7.0	——	——
	7.0–8.0	2.37	1.54–3.69
	>8.0	2.68	1.79–4.01

### Sensitivity analysis

Model 1, where participants of unknown diabetes status were categorized as not having diabetes, showed that risk of HBV infection was higher for people with diabetes relative to people without diabetes (RR 1.43, 95% CI 1.27–1.62;Table 4). A similar but stronger effect was observed in Model 2 (RR 2.51, 95% CI 2.14–2.94), in which participants of unknown status were categorized as diabetic.

**Table 4:** Sensitivity analysis to determine risk of new HBV infection after sample stratification based on age or diabetes status

Age, years	Diabetes	Model 1^1^		Model 2^1^	
		RR	95% CI	RR	95% CI
15–59	No	——	——	——	——
	Yes	1.45	1.18–1.78	2.42	1.87–3.13
≥60	No	——	——	——	——
	Yes	1.37	1.17–1.62	2.63	2.15–3.12
All	No	——	——	——	——
	Yes	1.43	1.27–1.62	2.51	2.14–2.94

1 To calculate results in Model 1, individuals with unknown diabetes status were classified as not having diabetes; in Model 2, individuals with unknown status were classified as having diabetes.

## Discussion

This large population-based study in China suggests that people diagnosed with diabetes are at approximately 1.5-times higher risk of HBV infection than people not diagnosed with diabetes. This increased risk persisted in sensitivity analyses that assessed the possible influence of unknown diabetes status in our sample. The large population in the present study allowed us to control for demographic characteristics in order to obtain more accurate estimates of the risk of HBV infection.

The present study is the first to evaluate the risk for HBV infection amongst people with diagnosed diabetes in China, and our findings are consistent with results from two previous epidemiological studies in the U.S. A national representative survey showed that HBV infection was 60% more prevalent amongst people with diagnosed diabetes than amongst people without diagnosed diabetes between 1999 and 2010 [30]. In another U.S. study, comparison of 865 patients with acute hepatitis B and 90941 controls found that diabetic adults without hepatitis B risk behaviors were 1.9-times more likely to have acute hepatitis B than non-diabetic adults [31]. In Turkey, comparison of 630 people with diabetes and 314 without diabetes found significantly positive associations of HBsAg-positive serology with history of hospital admission, longer duration of diabetes, and use of insulin [32].

HBV outbreaks amongst people with diabetes have a long but underappreciated history. The earliest reports, from Sweden in 1926 and England in 1943–1945, described outbreaks of jaundice, then serum hepatitis, and finally HBV infection amongst patients visiting diabetes clinics for blood glucose monitoring [33,34]. The mode of HBV transmission in these studies was identified to be blood-contaminated equipment used by multiple patients. The first report of HBV transmission associated with fingerstick devices in the U.S. was in 1990 [35]. Studies in other countries have also reported outbreaks of acute hepatitis B amongst people undergoing blood glucose monitoring, primarily in institutional care settings such as nursing homes and assisted-living facilities [36,37]. HBV transmission in these studies occurred primarily due to lapses in infection control, such as poor hand hygiene or use of individual diabetes care devices on multiple people. Why diabetes should predispose individuals to HBV infection remains unclear and should be investigated in future studies.

Our results revealed an increased risk of HBV infection amongst diabetic individuals aged ≥60 years, consistent with a previous study documenting HBV outbreaks amongst older adults in institutional settings, such as long-term care facilities. We attribute the higher HBV risk in such individuals to their more frequent use of medical resources and generally poorer immune function [24].

Furthermore, our study found that individuals with higher fasting blood glucose were at increased risk of HBV infection, this is in agreement with a previous study from Yemen [38]. Diabetes mellitus leads to the disruption of multiple organ functions, including the suppression of endocrine function, impaired cellular immunity, and hyperhumoral immunity [19]. Long-term continuous hyperglycemia has been shown to accelerate the occurrence and development of chronic complications of diabetes, such as retinopathy, neuropathy, and immune disorders [39]. In addition, immunocompromised patients are at higher risk of HBV infection than individuals with normal immune systems [17]. Therefore, we hypothesize that diabetic patients may be at increased risk of HBV infection due to impaired immune function. The relationship between immune function and risk of HBV infection in diabetic patients will be addressed in future studies. Regardless of this relationship, active treatment and adequate blood glucose control in diabetic patients are required to significantly improve their quality of life and prolong their lifespan.

China is considered a highly endemic country for HBV, and the incidence of the infection in our control group was 3.8%. According to the seroepidemiological survey of the Chinese population in 2006, the HBsAg carrier rate of the population aged 1–59 years was 7.18%, making an estimated number of 93 million HBsAg carriers in the country [2]. The direct economic loss caused by viral hepatitis is estimated to be at least 50 billion yuan every year [40]. HBV infection not only causes serious health damage to patients and heavy economic burden on families and society, but also causes a series of social problems, which have become a major public health problem to be solved urgently in China [40].

Vaccination against HBV is the most effective way to prevent and control transmission [1]. In our study, 6.04% of newly HBV-infected patients with diabetes reported that they had received at least one dose of HBV vaccine, compared with 11.05% of newly infected people without diabetes. Our results support the effectiveness of HBV vaccination, even though most of our study participants received only one dose of vaccine. Although newborn HBV vaccination has been integrated into the Expanded Program on Immunization in China, it is not currently being implemented nationwide because of resource limitations [41]. In October 2011, the Advisory Committee on Immunization Practices recommended HBV vaccination for unvaccinated adults with diabetes aged 19–59 years and, at the physician’s discretion, for diabetic adults aged ≥60 years. People diagnosed with diabetes mellitus should receive HBV vaccination as soon as possible, since vaccination of young adults with diabetes leads to similar protection rates as amongst people without diabetes, and because the immune response elicited by HBV vaccination has been shown to decline with age and comorbidities [42–44].

The present study had several limitations. First, we based the classification of new HBV infections on HbsAg positivity, since data on acute hepatitis B were unavailable. Second, our data on exposure history and history of HBV vaccination were based on self-reporting, creating risk of recall bias. Third, several known risk factors of HBV infection were not investigated in the present study, such as injection drug use, sex between men, or sex with multiple partners. Fourth, we did not have data on frequency of blood glucose monitoring performed by a healthcare professional.

Despite these limitations, our findings suggest an increased risk of HBV infection amongst people diagnosed with diabetes mellitus in China. HBV vaccination and continuous infection control practices during the management of individuals with diabetes may help to reduce HBV transmission in this vulnerable population.

## Abbreviations

CI: confidence interval
ELISA: enzyme-linked immunosorbent assay
GDP: Gross Domestic Product
HBsAg: hepatitis B surface antigen
HBV: hepatitis B virus
RR: relative risk
